# Association of pain and CNS structural changes after spinal cord injury

**DOI:** 10.1038/srep18534

**Published:** 2016-01-06

**Authors:** Catherine R. Jutzeler, Eveline Huber, Martina F. Callaghan, Roger Luechinger, Armin Curt, John L. K. Kramer, Patrick Freund

**Affiliations:** 1 Spinal Cord Injury Center, University Hospital Balgrist, University of Zurich, Zurich, Switzerland; 2 Faculty of Education, School of Kinesiology, ICORD, University of British Columbia; 3 Institute for Biomedical Engineering, University and ETH Zurich, Zurich, Switzerland; 4 Department of Brain Repair and Rehabilitation, UCL Institute of Neurology, University College London, London, UK; 5 Wellcome Trust Centre for Neuroimaging, UCL Institute of Neurology, University College London, London, UK; 6 Department of Neurophysics, Max Planck Institute for Human Cognitive and Brain Sciences, Leipzig, Germany

## Abstract

Traumatic spinal cord injury (SCI) has been shown to trigger structural atrophic changes within the spinal cord and brain. However, the relationship between structural changes and magnitude of neuropathic pain (NP) remains incompletely understood. Voxel-wise analysis of anatomical magnetic resonance imaging data provided information on cross-sectional cervical cord area and volumetric brain changes in 30 individuals with chronic traumatic SCI and 31 healthy controls. Participants were clinically assessed including neurological examination and pain questionnaire. Compared to controls, individuals with SCI exhibited decreased cord area, reduced grey matter (GM) volumes in anterior cingulate cortex (ACC), left insula, left secondary somatosensory cortex, bilateral thalamus, and decreased white matter volumes in pyramids and left internal capsule. The presence of NP was related with smaller cord area, increased GM in left ACC and right M1, and decreased GM in right primary somatosensory cortex and thalamus. Greater GM volume in M1 was associated with amount of NP. Below-level NP-associated structural changes in the spinal cord and brain can be discerned from trauma-induced consequences of SCI. The directionality of these relationships reveals specific changes across the neuroaxis (i.e., atrophic changes versus increases in volume) and may provide substrates of underlying neural mechanisms in the development of NP.

Traumatic spinal cord injury (SCI) is thought to drive structural changes–both degeneration and repair–across the entire neuroaxis (brain and spinal cord)^1^,^2^. Neuroimaging studies have demonstrated a relationship between the extent of the plasticity and the recovery of sensorimotor impairments, proposing a functional rationale for “reorganization” after SCI^3^,^4^,^5^. A major limiting factor of the recovery is the development of below-level neuropathic pain (NP) of which the majority of the individuals with SCI suffer from^6^,^7^,^8^,^9^. Commonly, the development of below-level NP in SCI has been attributed to maladaptive plasticity in brain areas encoding sensory stimuli (i.e., noxious and innocuous sensation) including primary and sensory cortices (S1 and S2), thalamus, and anterior cingulate cortex (ACC)^10^,^11^. Cortical reorganization of primary somatosensory cortex (S1) was shown to be associated with the intensity and duration of ongoing NP following SCI^11^,^12^. These findings prompt the assumption that persistent NP is related to alterations in neuronal activity that translate into long-term structural changes in the brain^12^,^13^. Gray and white matter volume changes were detected in SCI individuals suffering from NP when compared to pain-free counterparts^10^. Trauma-induced cord atrophy rostral to the injury is frequently reported^5^,^14^, however less is known about the pathological relationship between cord atrophy and below-level NP.

The primary objective of this study was to investigate the impact of traumatic SCI on central nervous system (CNS) structure, and the extent by any which changes in structure were related to the presence of below-level neuropathic pain. Building on previous studies that focused exclusively on the brain, we aimed to address the hypothesis that below-level neuropathic pain is indicative of structural changes in the spinal cord compared to pain-free individuals and healthy control subjects. Among individuals with neuropathic pain, we further aimed to examine the direction of the relationship between changes in CNS structure and the intensity of patient reported symptoms. Thus, we used cross-sectional cord area measurement to assess cord atrophy and voxel-based morphometry/thickness to assess gray and white matter volumes changes^5^. We specifically addressed the hypothesis that structural changes across the neuroaxis are associated with the presence and intensity of below-level NP. To this end, we investigated healthy subjects, individuals with SCI with NP, and pain-free individuals with SCI.

## Material and Methods

### Data collection

#### Participants

We enrolled 30 individuals with a chronic traumatic SCI (mean (SD) 46.3 (11.9) years; gender: 3 female, 27 male) including lesions at cervical (N = 15), thoracic (N = 13), and lumbar levels (N = 2). Time since injury spanned from 2 to 27 years (mean 10.5 yrs) and 2 to 26 years (mean 13 yrs) for the individuals with tetraplegia and paraplegia, respectively. We also recruited 31 neurologically healthy subjects (mean (SD) 42.1 (9.9) years; gender: 8 female, 23 male). All participants provided written informed consent and all procedures described below were in accordance with the Declaration of Helsinki and approved by the local Ethics committee of Zurich the ‘*Kantonale Ethikkommission Zurich, KEK’* (ref. number: EK-04/2006).

### Clinical Assessment

The neurological examination was performed according to the International Standards for Neurological Classification of Spinal Cord Injury (ISNCSCI)^15^,^16^. Briefly, sensory, motor, and neurological levels of injury were identified allowing characterization of sensory/ motor functioning as well as determination of the completeness of injury by means of the ISNCSCI Impairment Scale (AIS)^16^. Sensory levels are assessed by testing two aspects of sensation, light touch and pin prick (sharp-dull discrimination), of a keypoint in each dermatome (C4-S4-5, bilateral). Motor function assessment comprised testing key muscle functions corresponding to 10 paired myotomes (C5-T1 and L2-S1). A ‘Touch-test Sensory Evaluator’ (North Coast Medical Inc, California, USA) was used for the evaluation of the cutaneous sensation level (Von Frey Filament testing) of the participants’ left and right C6 dermatomes. Beginning with the smallest filament size (2.83), the stimulus was applied three times, whereas a single response indicated a positive response. In cases in which the participant did not respond to the stimulus, the next largest monofilament was chosen and the process repeated. At maximum, a set of five of monofilaments was used (2.83, 3.61, 4.31, 4.56, and 6.65). Moreover, warm perception and pain thresholds of left and right C6 dermatomes were recorded using the method of levels starting at a baseline temperature of 32 °C^17^. Briefly, single stimuli with increasing temperature (1 °C/ stimulus) were sequentially presented by a contact heat stimulator (Pathway, Medoc, Ramat Yishai, Israel) until the subject reported to perceive a thermal sensation (i.e., perception threshold) or rated the thermal sensation as painful (i.e., pain perception). All participants were interviewed to determine the existence of pain using the EMSCI pain questionnaire (V4.2, http://www.emsci.org/). The pain questionnaire examines various aspects of pain (e.g., duration (years), maximal and average pain intensity). The pain intensity was rated using an 11-point numeric rating scale with “0” indicating no pain to “10” indicating worst pain imaginable. Accordingly, pain can be grouped into nociceptive (e.g., musculoskeletal or visceral) or neuropathic pain (e.g., at or below the lesion). To be classified as below-level NP, ongoing pain had to be located three of more segments below the level of lesion.

### Image acquisition

MRI data was collected on a Philips 3 T Ingenia system (Philips Medical Systems, Best, the Netherlands) using a 15-channel Philips Sense head coil. A 3D-GRE T1-weighted (T1w) sequence was used to acquire a structural scan optimized for simultaneous assessment of the brain and spinal cord^4^. The imaging parameters were: isotropic 1 mm^3^ resolution, field of view 256 × 256 × 180, repetition time = 6.88 ms, echo time = 3.1 ms, flip angle 8°, fat saturation, scan resolution 256 × 256 voxels, and a scan time of 6:31 min. Prior to analysis the MRI data were screened for movement artefacts.

### Data Analysis

#### Statistics

All statistical procedures were performed using SPSS (version 19.0, Armonk, New York, U.S.). Mann-Whitney-U tests were applied and P < 0.05 was considered significant after Bonferroni correction.

### MRI Analysis

Voxel-based morphometry (VBM) was performed in SPM8 in order to perform voxel-wise comparisons of gray (GM) and white matter (WM) volume between the three groups of subjects^18^. The preprocessing included four steps. Firstly, all 3D-GRE T1-weighted images were reoriented to set the image origin (0/0/0) to the anterior commissure. Then, a unified model inversion (unified segmentation) was used for bias correction and segmentation of 3D-GRE T1-weighted (T1w) images into GM, WM, and CSF. In the next step, through iterative nonlinear registration (DARTEL) the GM and WM segments were warped into an optimal (average) space. The resulting GM and WM images were modulated and affine transformed to MNI space. Lastly, the modulated normalized GM and WM segments were smoothed using an isotropic Gaussian kernel with 6 mm full width at half-maximum prior to between-group analyses at the 2nd level.

### Voxel-based cortical thickness

A voxel-based cortical thickness (VBCT) map was created for each participant using the GM, WM, and CSF segments created in the preprocessing step of the VBM analysis^19^. The input tissue segments were sub-sampled from 1 mm to 0.5 mm using trilinear interpolation to increase resolution for narrow CSF spaces. In order to compute VBCT maps, cortical GM boundaries were extracted and the distance between the inner and outer GM boundaries were estimated for each voxel in the cortex^19^. The VBCT maps comprised of a value for cortical thickness at each voxel in GM and zeros elsewhere. Afterwards, the VBCT maps were warped into the same reference space as the GM probability maps. Subsequently, smoothing was performed by using an isotropic 6 mm full width at half-maximum Gaussian kernel including a correction in order to maintain local cortical thickness^19^.

### Region of interest (ROI) Analysis

Based on previous neuroimaging evidence^10^,^11^,^14^,^20^,^21^,^22^, we chose an ROI approach incorporating the bilateral primary motor cortex (M1), somatosensory cortices (S1 and S2), premotor cortex (PMC), insulae, thalamus, and anterior cingulate cortex (ACC). ROIs and the white matter mask were created using the Pick Atlas in SPM8^23^,^24^. The John Hopkins University (JHU) White-Matter Tractography Atlas was used to identify the location of significant peak voxels in the white matter^25^. The ROIs were included as a mask in order to restrict the voxel-by-voxel analysis to these distinct regions. Group comparisons were performed between healthy controls and individuals with SCI, as well as between individuals with SCI suffering from NP and pain-free individuals with SCI. All results reported are family-wise error (FWE) corrected for multiple comparisons within regions of interest. For all group (i.e., healthy controls and individuals with SCI) and subgroup (i.e., pain-free individuals with SCI and individuals with SCI suffering from below-level NP) analyses, we accounted for the variability between individuals with SCI and healthy volunteers by explicitly modeling the linear variance of age, sex, level of lesion, and total intracranial volume (TIV) in all GLM analyses^26^. Then we extracted the contrast estimates of all ROIs from the individuals with SCI in order to perform Spearman correlations to identify associations between structural changes and clinical characteristics (i.e., SCA, ISNCSCI motor and sensory scores, disease duration, neurological level of lesion, pinprick and light touch scores, pain intensity and duration)^27^.

### Spinal Cord Area (SCA), anterior-posterior width (APW), and left-right width (LRW)

The 3D-GRE T1-weighted images were used to identify the cross-sectional cord area based on a well-established semi-automated segmentation method^5^,^20^,^28^. User intervention was required to define the cervical vertebra C2 as an anatomic landmark and to define the center of C2 on the axial slice containing the anatomic landmark voxel. This landmark definition was performed by an expert in spinal cord imaging, CRJ. The remaining analysis was carried out automatically to minimize the risk of user-induced bias. The boundary of the cord was determined via nearest-neighbor region growing. The stopping criterion was a 25% drop in signal intensity relative to the mean of the region. This was taken to signify that the white matter-cerebrospinal fluid boundary had been reached. The boundary voxels of this region were then used to fit an ellipse from which the anterior-posterior width (APW, long axis of the ellipse), left-right width (LRW, short axis of the ellipse) and cord area were extracted. The quality of the fit was ensured by visual inspection (also performed by CRJ). The center of the region of interest (ROI) was used to initialize region growing in the adjacent superior slice. A total of 15 slices were included in the analysis giving a total caudal-rostral coverage of 1.5 cm. The average of APW, LRW and cord area over this 1.5 cm extent was used for statistical analysis. The Kruskal–Wallis one-way analysis of variance was used to identify differences in cord area, APW, and LRW between controls and subjects with SCI. In individuals with SCI, regression analyses were performed to test for an association between impairment and spinal cord area, APW, and LRW. The behavioral measure was set as the dependent variable, while spinal cord area, APW, and LRW were included as independent variables. Age and gender are reported to have a significant effect on the spinal cord area^29^, both variables were included as covariates in the analysis in addition to the lesion level.

## Results

### Clinical measurements

According to the ISNCSCI Impairment classification, 12 of 30 SCI subjects had complete (2 tetraplegic, 10 paraplegic) and 18 incomplete (13 tetraplegic, 5 paraplegic) injury of the spinal cord. Two SCI subjects (1 individual with tetraplegia, 1 with paraplegia) were excluded due to incomplete datasets (i.e., measurement errors). All individuals with SCI had reduced ISNCSCI motor and sensory scores of upper and lower limbs. From the remaining 28 individuals with SCI, 13 (8 with paraplegia, 5 with tetraplegia) suffered from below-level NP. Only one individual with SCI suffered from both at- and below-level pain (Table 1).

The average and maximal pain intensities were 4.0 ± 2.1 and 5.5 ± 3.0, respectively, and the duration of ongoing pain ranged from 4 to 33 years (mean 19.8 ± 12.3). All other individuals were pain-free at the time-points of measurements. Individuals with SCI showed elevation of thermal perception thresholds (paraplegic SCI: F = 13.4, df:56, p = 0.010, tetraplegic SCI: F = 13.4, df:56, p < 0.001) and pain thresholds (paraplegic SCI: F = 14.39, df:56, p < 0.001; tetraplegic SCI: F = 15.1, df: 56 , p = 0.003) compared to healthy controls (Table 2).

### Structural changes at spinal cord and brain

**Figure 1:**
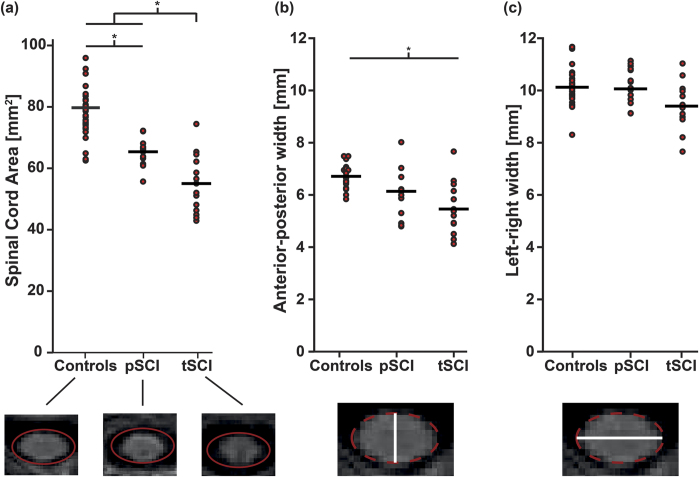
Spinal cord area reduction as a consequence of SCI. (**a**) A significant reduction in SCA was found in individuals with paraplegia (pSCI) and tetraplegia (tSCI). The degree of reduction was more pronounced in individuals with tetraplegia. (**b**) In individuals with tetraplegia, a significant decrease of the anterior-posterior width is observed compared to healthy controls, while (**c**) the left-right width is indifferent between the groups.

Overall, cross-sectional SCA was reduced by 25.4% (mean (SD) 79.2 ± 9.1; F = 33.231, df: 56, p < 0.001) in the SCI cohort compared to healthy controls (i.e., 17.2% (6 ± 5.1; F = 50.64, df:42, p < 0.001) paraplegic SCI; 27.2% (56.0 ± 10.2; F = 22.81, df:42, p < 0.001) tetraplegic SCI). The degree of cord atrophy in SCI individuals with tetraplegia was greater than in individuals with paraplegia −14.0%; F = 9.0, df:26, p = 0.015) (Fig. 1 and Table 1). Differences in anterior-posterior width (APW) were only observed in individuals with tetraplegia (−18.2%; F = 23.3, df:42, p = 0.001) when compared to healthy controls. No differences between groups were detected regarding left-right width (LRW) (p > 0.05). Lastly, no difference between individuals with incomplete and complete SCI were found (p > 0.05).

At the whole brain level analysis, no group differences of GM and WM cortical volumes were found (all p > 0.05). However, employing the ROI approach revealed decreased GM and WM cortical volumes in individuals with SCI compared to healthy controls (Table 3). Specifically, lower GM volumes were found in ACC (Z score = 3.73, p = 0.047), left insula (Z score = 4.25, p = 0.002), left secondary somatosensory cortex (Z score = 4.83, p = 0.002 and Z score = 3.96, p = 0.009), and bilateral thalamus (Z score = 3.71, p = 0.031 (left) and Z score = 3.94, p = 0.045 (right)). White matter volumes were decreased in the pyramids (Z score = 3.98, p = 0.009, (left) and Z score = 3.83, p = 0.015 (right)) and the left internal capsule (Z score = 4.12, p = 0.021) in individuals with SCI. Similar to and beyond the area identified by VBM, VBCT analysis revealed decreased cortical thickness in left secondary somatosensory cortex (Z score = 4.01, p = 0.024) in subjects with SCI compared with controls. The cluster, containing the significant peak voxel extended in a rostral–caudal direction from y = −49.5 to −64.5, in the ventral–dorsal direction from z = 9 to 31.5 and left-right direction x = −9 to −21, thus additionally encompassing left M1 and right S1. Employing the whole brain analysis and ROI approach, no differences between individuals with complete and incomplete SCI were identified.

### Relationship between below-level neuropathic pain and structural changes across the neuroaxis

**Figure 2:**
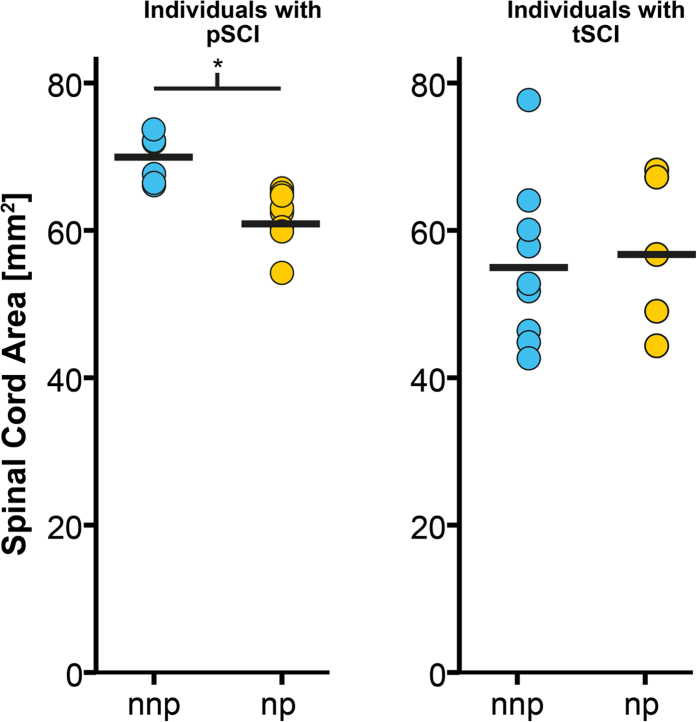
The relationship between the presence of below-level neuropathic pain (np) and spinal cord atrophy. Subjects with paraplegia (pSCI) suffering from neuropathic pain (np) exhibit lower cord area compared to their pain-free counterparts (nnp). Such a difference was not observed in subjects with tetraplegia (tSCI).

**Figure 3:**
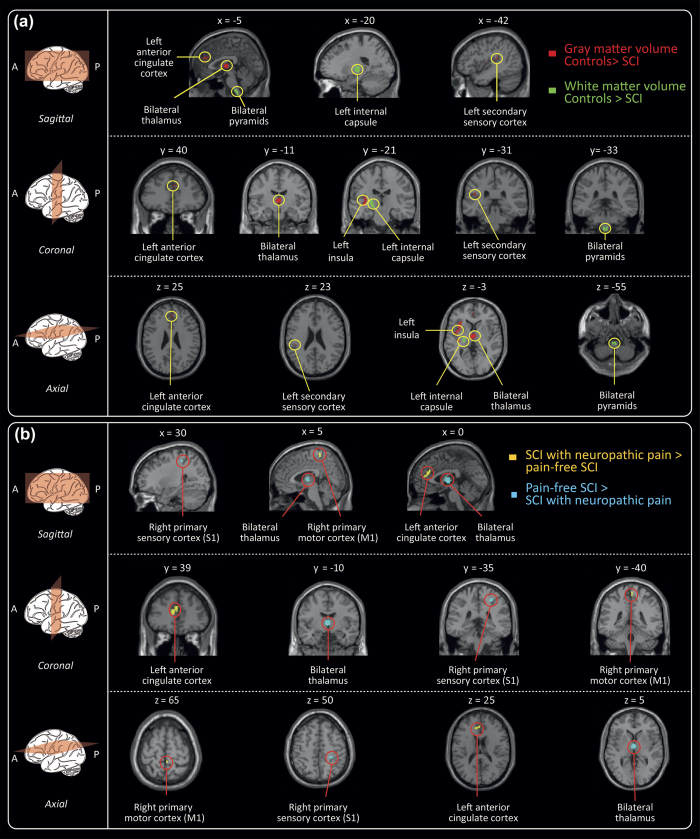
Trauma-induced and pain-related changes of the cerebral structure. (**a**) Volumetric changes between healthy subjects (HC) and individuals with SCI. Individuals with SCI exhibit decreased gray matter volume (red) in the left anterior cingulate cortex, left insula, left secondary sensory cortex, as well as bilateral thalamus compared to healthy controls. Further, decreased white matter volumes (green) were detected in bilateral pyramids and left internal capsule. (**b**) Bidirectional pain-related morphological changes in individuals with SCI experiencing neuropathic pain. In comparison to their pain-free counterparts, spinally injured individuals with neuropathic pain exhibit decreased gray matter volumes (blue) in the right primary sensory cortex (S1) and in the thalamus. In pain-free individuals with SCI, smaller gray matter volumes (yellow) were observed in the right primary motor cortex and the left anterior cingulate cortex compared to the individuals with SCI suffering from neuropathic pain. All volumetric changes displayed are derived from ROI analyses.

**Figure 4:**
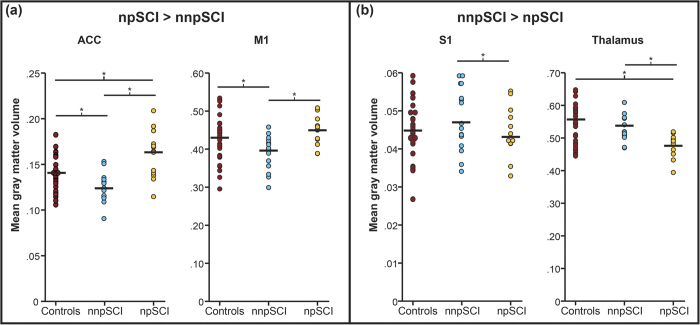
Mean tissue volumes in SCI Individuals with (npSCI) and without neuropathic pain (nnpSCI) compared with healthy control individuals. Averaged ROI volumes extracted for the supra-threshold clusters identified from whole brain contrasts (p < 0.001 uncorrected) in Table 4. (**A**) Decreased volumes were found in pain-free individuals with SCI in the anterior cingulate cortex (ACC) and primary motor cortex (M1) compared to the pain-free SCI group. Surprisingly, the SCI-NP group exhibited even greater volumes in the ACC compared to healthy individuals. (**B**) Pain-free individuals with SCI displayed lager gray matter volumes in the primary sensory cortex (S1) as well in the thalamus. All extracted volumes were corrected for age and gender prior to extraction. npSCI: Individuals with SCI suffering from neuropathic pain. nnpSCI: Pain-free individuals with SCI.

**Figure 5:**
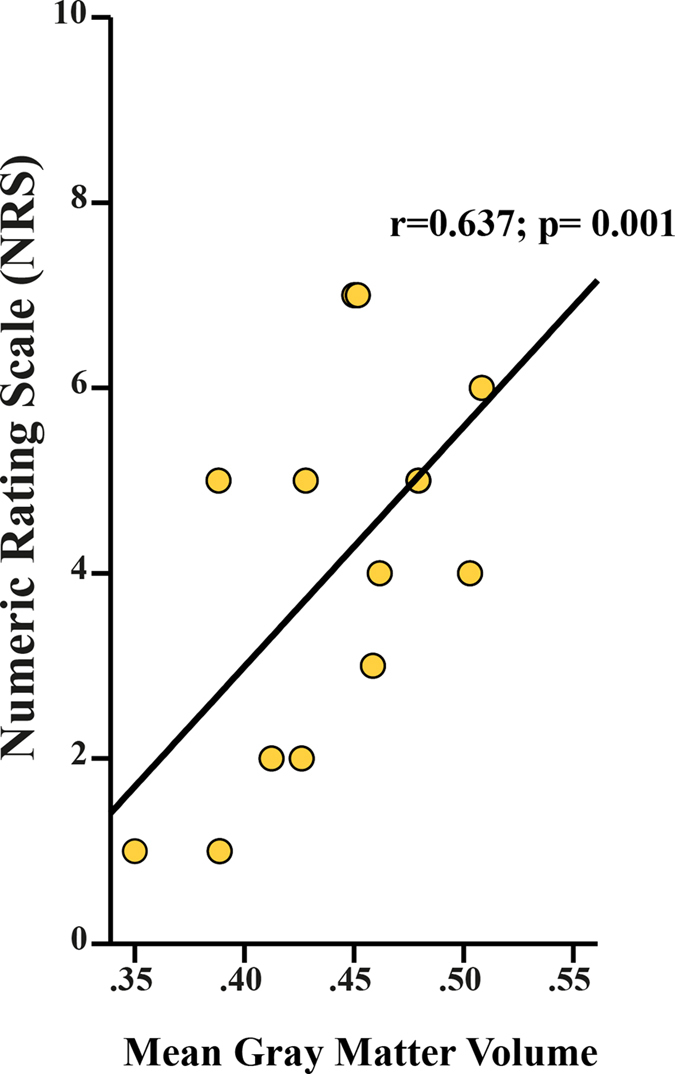
Positive correlation between preserved local gray matter volume and ongoing pain intensity. Greater gray matter volume in primary motor cortex (M1) is associated with higher on-going pain intensity in individuals with SCI suffering from below-level neuropathic pain.

Individuals with paraplegia suffering from NP, but not the individuals with tetraplegia (p > 0.05), exhibited a more decreased cord area compared to their pain-free counterparts (p = 0.002, greater mean reduction of −11.6%) (Fig. 2). At the spinal level, cord area was associated with neurological level of lesion (r = 0.558, p = 0.020) and pathologically increased warm perception threshold (r = −0.486, p = 0.049) – that is, warm perception thresholds were increased with smaller SCA. Cord area changes were independent of severity of injury (ISNSCI Score) (r = −0.108, p = 0.583), time since injury (r = −0.103, p = 0.600), and age (r = −0.091, p = 0.644). At the brain level, a decrease in GM volumes in the left ACC (Z = 3.85, p = 0.034) and right M1 (Z = 4.33, p = 0.005) was detected in NP-free individuals with SCI when compared to individuals suffering from below-level NP. On the other hand, NP-free individuals with SCI show greater GM volumes in the right S1 (Z = 3.79, p = 0.046), and thalamus (Z = 4.16, p = 0.010) (Table 4) as well as Fig. 3 and 4). In addition, greater GM volume in M1 is associated with higher on-going pain intensity in individuals with SCI suffering from NP (r = 0.637; p = 0.001)(Fig. 5). No further correlations between anatomical changes and clinical outcomes were detected.

## Discussion

This study shows distinct association of below-level NP with structural changes of the spinal cord and brain after SCI. In individuals with paraplegia, the reduction in SCA was associated with below-level NP independent of the level or completeness of lesion. At the brain level, pain-related changes were of bi-directional nature (i.e., increased and decreased volumes) and located in areas relevant to nociceptive processing (i.e., including S2, insula, ACC, thalamus) as well as in brain areas not associated with the sensation of NP (i.e., S1, M1, pyramids, internal capsule). These findings provide further insights into the complex pattern of neuro-adaptive processes due to below-level NP following SCI.

### Below-level neuropathic pain and structural changes of the spinal cord

While several studies provided evidence of trauma-induced atrophy of the spinal cord^5^,^14^,^30^ and its relation to clinical recovery and impairment, this is the first study to demonstrate an association between spinal cord atrophy and the presence of neuropathic pain. The magnitude of cord atrophy above the neurological level of lesion (all measures performed at C2) was associated with the presence of below-level NP in individuals with paraplegia. That is, individuals with greater cord atrophy were more likely to exhibit below-level NP independent of the neurological level of lesion. Due to the cross-sectional nature of this study, the question of whether below-level NP is causal or consequential cannot be addressed. Based on recent findings, cord atrophy is considered to be due to anterograde and retrograde axonal degeneration of spinal pathway^31^.

However, pain might arise from both the abolishment of specific fiber tracts reflected by atrophic changes, but also changes through aberrant sprouting of spinal circuits may cause disinhibition or imbalance between sensory and motor pathways^32^. On the contrary, degeneration of myelinated efferent fibers is sufficient to elicit spontaneous activity in uninjured C-fiber afferents and has been associated with hyperalgesia and allodynia^33^. Another key factor in individuals with SCI might result from reduced neuro-muscular activity evident in individuals with chronic pain^34^. Typically, individuals suffering from pain tend to be less active due to the ongoing pain and its concomitants (e.g., depression, lack of motivation) or side-effects of pain medication. Increased activity promotes plasticity at multiple levels of the neuroaxis including spinal cord circuitry rostral and caudal to injury^35^. Thus, pain-related neuro-muscular inactivity leading to lower brain-derived neutrophic factor (BDNF) and tyrosine kinase B (TrkB) levels might inhibit these plastic processes resulting in atrophic structures as seen in the present study^36^. Up-regulated BDNF levels following intense exercise were associated with neuronal plasticity in skeletal muscles but also in the innervating level of the spinal cord^37^,^38^.

No such difference in the magnitude of cord atrophy related to pain was observed in individuals with tetraplegia. A potential reason might be a ‘flooring effect’ of atrophic progression towards a ‘stable’ SCA^3^,^5^. Cord atrophy rostral to the injury (i.e., C2 vertebrae) is dependent on the neurological level of lesion, as shown in the present study, – with more pronounced changes at higher levels of lesion^5^. Notably, given the anatomical properties of the spinal cord, a lesion to the cervical cord impacts the structural integrity of a higher number of fibers and neurons than a comparable thoracic lesion. The level-dependent degree of atrophy suggests axonal degeneration inducing cord atrophy as the morphometric changes were predominantly in the anterior-posterior axis of the cord. Changes in the anterior-posterior axis might represent Wallerian degeneration of afferents running in the dorsal columns^5^.

### Below-level neuropathic pain and structural changes of the brain

In individuals with SCI, below-level NP was accompanied with reduced GM volume in S1 and the thalamus, but increased GM in the M1 and ACC. Thus far, several VBM studies have reported morphological alterations in areas related to pain processing in patients with neuropathic pain^10^,^39^, phantom pain^20^,^22^,^40^, fibromyalgia^41^,^42^, and migraine^43^,^44^,^45^ (findings summarized in Table 5). Volumetric bi-directional alterations of GM in areas involved in processing afferent input (e.g., thalamus, insula, cingulate cortex) have been reported^46^. While, decreased GM volume in pain-regulating areas is often put in context with maladaptive structural plasticity^46^,^47^, increased GM is rather linked with preserved structure as the underlying source of chronic pain^22^. ‘Maladaptive plasticity’ predicts that following loss of efferent output and afferent input deprived central areas undergo structural alterations of synaptic connections thereby triggering NP. The neuronal substrates underlying injury-induced alterations remain incompletely understood. VBM is sensitive to structural volume changes (i.e., alterations in WM and GM), but it cannot disclose the neuronal substrates underlying the structural differences that are revealed in the present study and thus assumptions regarding the underlying neuronal substrates remain speculative. Alterations in GM could result from varying numbers of neurons, interneurons, or glia cells, but also by differently sized cells. Based on animal studies, proposed mechanisms for the volume alterations include axonal sprouting, neuronal death, synaptic pruning, and activity-dependent plasticity^48^. Moore and colleagues proposed in their pre-clinical study that a decline in GM, potentially due to persistent nociceptive input to the brain, could result in neural hyperexcitability along the neuroaxis^49^. Albeit incomplete understanding of the exact genesis of these morphological changes, it is yet generally accepted that chronic pain conditions are associated with alterations of gray matter volume localized in pain processing and modulatory areas, such as thalamus, insula, and anterior cingulate cortex. As such, morphometric alteration in the thalamus, as seen in the present study, is a robust and consistent observation across various pain conditions^42^,^47^,^50^. The thalamus, a major relay station of sensory information, has been implicated in pain modulation and its dysfunction is suspected to drive the pathogenesis of various pain disorders (e.g., NP, fibromyalgia, migraine)^46^. In fact, human and animal studies provide evidence for an involvement of the thalamus in the emergence of neuropathic pain^51^,^52^,^53^,^54^,^55^,^56^ manifested as reorganization of the thalamus and abnormal thalamic rhythmicity. Anatomical deafferentation is likely the underlying mechanism resulting in a general thalamic hypoactivity (e.g., deficit in thalamic metabolism, decreased thalamic blood flow, functional thalamic depression, ‘thalamocortical dysrhythmia’) which eventually might become autonomous and contribute to the pathophysiology of NP^20^,^57^,^58^,^59^. Furthermore, the observed decreased GM volumes in primary somatosensory cortex is in line with previous studies associating structural and functional alterations with the presence of chronic pain following deafferentation^60^,^61^. Similar to the thalamus, S1 belongs to the so-called ‘lateral’ pain system and possesses a pivotal role in encoding sensory-discriminative aspects of pain^62^,^63^. Ongoing nociception was hypothesized to be associated with structural reorganization of areas involved in pain processing (e.g., thalamus and S1), which in turn may play a role in the process of the chronification of pain^64^. Thus, the decrease in GM volume in the thalamus and primary somatosensory cortex that we observed in individuals with SCI suffering from pain might reflect a chronic nociceptive transmission and may represent cortical plasticity.

In contrast to the maladaptive plasticity model, Mole and colleagues recently provided evidence that the presence of below-level neuropathic pain is associated with ‘preserved’ GM (i.e., GM volume comparable to healthy controls) in primary somatosensory and motor cortices^10^. This is in agreement with our findings, individuals *without* neuropathic pain demonstrate greater differences from healthy subjects than do individuals with neuropathic pain^10^. Similar to Makin^22^, we show not only the relationship of below-level NP and maintained local GM in M1, but also the correlation between pain intensity and preserved GM volume. Maintained local structure with disturbed long-range connectivity and diminished inhibition by surrounding representational areas may lead to ‘autonomous-acting’ areas responsible for the experience of pain^22^. The positive correlation with the intensity of ongoing below-level NP strengthens the assumption that pain is the consequence of structural preservation. We also found decreased GM volume in the anterior cingulate cortex (ACC) in pain-free individuals with SCI when compared to individuals suffering from neuropathic pain. This finding is somewhat surprising and challenges explanation, since numerous studies contrast our findings by reporting pain-related volume *decrease* in the ACC^20^,^43^,^46^. One possibility could be that the ongoing pain prevents deafferentation-related structural alteration. This would be in accordance with training studies revealing a positive correlation between intensity of activity and increase of gray matter in areas ascribable to the task^1^,^65^,^66^,^67^. Furthermore, the anatomical connections between ACC and M1 suggest that these regions might be interactive in encoding different aspects of pain^68^ and thus, the similarity of the regional volumetric changes (i.e., relative increase) in the presence of chronic pain are in line with this possibility.

Finally, in this cohort, structural alterations of the WM and cortical thickness were not related with pain, but with the trauma-induced deafferentation. Based on animal studies, retrograde axonal degeneration is also suspected to induce volumetric changes of WM tissue at multiple levels of the corticospinal tract^69^ – as in the present study in the pyramids and internal capsule. Lastly, cortical thinning of the secondary somatosensory cortex was detected in the SCI group compared to healthy controls indicating atrophy of neurons, glial cells, and/or extracellular space^70^,^71^,^72^. Again, no link with the ongoing below-level NP and degree of cortical thinning was found.

### Limitations

A major limiting factor constitutes that individuals with SCI and healthy volunteers are not matched with respect to gender. This can confound the group comparison, given possible differences in structure according to gender. DARTEL was used to improve the equalization between the groups through normalization processes. Furthermore, the cross-sectional nature of the study restricts conclusions to a single time point only and thus, temporal information of the above-described structural changes remain speculative. Outside the scope of this study, the assessment of depression and anxiety indices would be interesting to show that observed effects cannot be attributed to prevalent psychiatric comorbidity in this clinical group.

## Conclusion

We provide further evidence of extensive changes in sensory function (i.e., pain) and micro-structural morphology of the CNS rostral to the neurological level of lesion (spinal cord and brain). Besides the trauma-dependent changes also volumetric changes become discerned that are related to below-level NP with both decreases and increases. The CNS shows extensive and complex changes (multi-directional with decrease – increase) in response to an injury. These complex interactions need to be considered in interventional studies that often simultaneously may affect many modalities like sensor-motor function and pain syndromes^11^,^73^. Future investigations are required to better understand the role of below-level NP in the process of dynamic structural changes after spinal cord injury^14^. It is conceivable that neuro-imaging biomarkers are sensitive in detecting these complex pain related neuro-adaptive changes.

## Additional Information

**How to cite this article**: Jutzeler, C. R. *et al*. Association of pain and CNS structural changes after spinal cord injury. *Sci. Rep*. **6**, 18534; doi: 10.1038/srep18534 (2016).

## Figures and Tables

**Table 1:** Clinical data for the spinal cord injured individuals.

ID	Age [yrs]	Gender	Etiology of the injury	Time since Injury [yrs]	Level of lesion‡	AIS*	Motor (0-100)	Sensory (0–224)†	Neuropathic pain
*Pain-free Individuals with SCI*									
P02	50	m	Vehicle accident	9	Th4	B	50	200	no
P06	44	m	Gunshot	17	Th6	A	50	96	no
P08	38	m	Vehicle accident	16	Th8	A	50	104	no
P11	60	m	Vehicle accident	2	Th12	D	100	185	no
P12	50	m	Vehicle accident	4	Th11	A	60	148	no
P13	43	m	Vehicle accident	24	Th6	A	50	110	no
T01	25	m	Sports accident	2	C6/ C7	D	71	134	no
T02	40	m	Vehicle accident	20	C5	C	38	126	no
T03	67	m	Vehicle accident	11	C4	D	95	170	no
T04	53	m	Gun shot	10	C4	B	27	102	no
T06	40	m	Vehicle accident	19	C6	B	24	78	no
T07	51	m	Vehicle accident	27	C6/ C7	A	30	83	no
T08	35	m	Sports accident	5	C6/ C7	D	93	176	no
T10	24	m	Vehicle accident	7	C6/ C7	A	40	185	no
T13	69	m	Vehicle accident	10	C4/C5	C	79	200	no
*Individuals with SCI suffering from Neuropathic Pain*									
P01	50	m	Vehicle accident	7	Th4/Th5	A	50	90	Below-level
P03	35	m	Gunshot	12	Th4	B	50	144	Below-level
P04	48	m	Vehicle accident	16	Th6/7	A	50	112	Below-level
P05	44	m	Vehicle accident	26	Th7	B	50	168	Below-level
P07	29	m	Sports accident	14	Th11	A	50	128	Below-level
P09	29	f	Sports accident	5	L3	D	100	217	Below-level
P10	65	m	Vehicle accident	33	Th12	A	50	158	Below-level
P14	48	f	Hit by tree	19	L1	A	50	144	Below-level
T05	57	m	Vehicle accident	6	C6/ C7	D	96	202	Below-level
T09	40	m	Vehicle accident	10	C7	D	96	160	Below-level
T11	55	m	Vehicle accident	4	C3	D	89	104	Below-level
T12	65	m	Vehicle accident	10	C6	D	88	220	Below-level
T14	45	m	Vehicle accident	5	C2	D	100	224	Below-level

^‡^The level of lesion refers to the neurological level.

^*^ASIA impairment scale: A, no sensory or motor function is preserved; B, sensory function is preserved below the level of the injury, but there is no motor function; C,motor function is preserved below the neurological level, and more than half of the key muscles below the neurological level have a muscle grade of <3; D, motor function is preserved below the neurological level, and at least half of the key muscles below the neurological level have a muscle grade of >3.

^†^Sensory Score: Sum of segmental light touch and pinprick classifications.

**Table 2:** Main clinical and demographic findings from the cohorts studied.

Parameter	Groups			Significant pairwise comparisons (p < 0.05‡)
	Healthy Controls	tSCI	pSCI	
Gender [m:f]	23:8	14:0	12 :2	HC-tSCI (<0.001), HC-pSCI ( = 0.005)
Age [yrs]	42.1 ± 9.9	47.6 ± 14.5	45.2 ± 10.3	ns
Duration of SCI [yrs]	NA	10.5 ± 7.2	14.5 ± 8.9	ns
SCA [mm^2^]	79.2 ± 9.1	56.0 ± 10.2	65.1 ± 5.1	HC-tSCI (<0.001); HC-pSCI (<0.001); pSCI-tSCI ( = 0.015)
AP-Width [mm]	8.7 ± 1.2	5.4 ± 0.9	6.2 ± 1.5	HC-tSCI ( = 0.001)
LR-Width [mm]	9.8 ± 1.0	9.4 ± 1.0	10.2 ± 0.7	ns
Von Frey Filaments	14.9 ± 2.09	5.2 ± 3.1	15.2 ± 3.1	HC-tSCI (<0.001); HC-pSCI (<0.001)
Perception Threshold [°C]	38.7 ± 1.0	41.6 ± 2.6	39.3 ± 2.5	HC-tSCI (<0.001); pSCI-tSCI ( = 0.010)
Pain Threshold [°C]	51.4 ± 1.7	54.8 ± 0.2	49.2 ± 3.3	HC-tSCI ( = 0.003); HC-pSCI (<0.001); pSCI-tSCI (<0.001)

Results are displayed as mean ± standard deviation.

ns = not significant.

SCA = Spinal Cord Area; AP = Anterior-posterior width of the spinal cord area; LR = Left-right width of the spinal cord area.

HC = healthy controls, tSCI = Individuals with tetraplegia, pSCI = Individuals with paraplegia.

^‡^Bonferroni corrected.

**Table 3:**
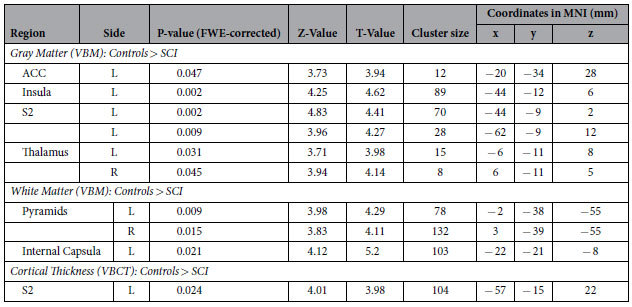
Regions of reduced gray and white matter as well as thinner cortical thickness in individuals with SCI compared to healthy controls.

FWE = Family wise error; ACC = Anterior cingulate cortex; S2 = secondary somatosensory cortex; L = Left; R = Right, MNI = Montreal Neurological Institute.

**Table 4:** Differences in gray matter volume between individuals with SCI suffering from neuropathic pain (npSCI) compared to pain-free individuals with SCI (nnpSCI).

Region	Side	P-value (FWE-corrected)	Z-Value	T-Value	Cluster size	Coordinates in MNI (mm)		
						x	y	z
*Gray Matter (VBM): npSCI > nnpSCI*								
M1	R	0.005	4.33	5.40	99	12	−32	59
ACC	L	0.034	3.85	4.58	46	−2	36	27
*Gray Matter (VBM): nnpSCI \ npSCI*								
Thalamus	L/R	0.010	4.16	5.10	152	0	−6	3
S1	R	0.046	3.79	4.01	27	24	−33	48

FWE = Family wise error; ACC = Anterior cingulate cortex; M1 = primary motor cortex; S1 = primary somatosensory cortex; L = Left; R = Right, MNI = Montreal Neurological Institute.

**Table 5:** Summary of VBM studies in chronic pain syndromes.

Study (year)	Study population	Type of pain (n = )	Summary of findings
Present study (2015)	28 individuals with complete/ incomplete SCI (cervical(14), thoracic (12), and lumbar (2)lesions); 31 healthy control subjects	Below-level neuropathic pain (13)	• Reduced gray matter volume of anterior cingulate cortex, insula, secondary sensory cortex, and thalamus in individuals with SCI compared to healthy controls • Among the SCI group, gray matter volume decrease of primary sensory cortex and thalamus were found in patients with pain compared to pain-free individuals with SCI. Gray matter volumes of primary motor cortex and anterior cingulate cortex were reduced in pain-free individuals with SCI compared to individuals with neuropathic pain. • Pain intensity was positively correlated with the gray matter volume of primary motor cortex
Draganski *et al*., (2006)	28 uni- or bilateral upper-/ lower arm and leg amputees (23 traumatic, 5 non-systemic); 20 age- and gender-matched healthy controls	Phantom-limb pain (28)	• Reduced gray matter in amputees compared to healthy controls • The thalamic gray matter differences were positively correlated with duration of amputation. • No relationship between pain intensity and gray matter volume.
Makin *et al*., (2013)	29 unilateral upper-limb amputees (18 traumatic, 11 congenital unilateral upper-limb deficit); 22 healthy controls	Phantom-limb pain (17)	• Reduced gray matter volume in amputees compared to healthy controls • Within the amputation group, gray matter volume was positively correlated with pain intensity
Preißler *et al*., (2013)	21 right upper limb amputees (19 traumatic, 2 sarcoma); 14 age-matched healthy controls	Phantom-limb pain (21)	• In comparison to healthy controls, amputees showed a significant increase in gray matter volume in the left temporal pole, left dorsolateral prefrontal cortex, left fusiform cortex, right middle temporal cortex and the right superior parietal cortex. Reduced gray matter was found in the primary motor cortex and the right dorsolateral prefrontal cortex • Within the patients group, amputees with high intensity pain showed reduced gray matter in brain areas involved in pain processing (anterior cingulate cortex, left motor cortex, superior temporal lobe). • Patients with low intensity pain showed a significant gray matter increase in regions of the visual stream. • Positive correlation between gray matter volume in the caudal anterior cingulate cortex and pain rating, negative correlation in the insular gray matter
Mole *et al*., (2014)	30 patients (complete/incomplete 21 cervical or 9 thoracic lesions); 18 age- and gender-matched healthy controls	Below-level neuropathic pain (18)	• Pain-free SCI individual showed increase in gray matter volume in primary sensory cortex. No difference in gray matter volume was found between SCI individuals with neuropathic pain compared to healthy controls • Amongst individuals with SCI suffering from neuropathic pain, primary sensory cortex gray matter volume was negatively correlated with pain intensity
Yoon *et al*., (2003)	10 patients (5 motorcomplete cervical, 5 motorcomplete thoracic lesions), 10 age- and gender-matched healthy controls	Neuropathic pain (10)	• The patients showed significant reductions in gray matter volume of left middle frontal gyrus, bilateral anterior insulae, and right subgenual anterior cingulate cortex
Rocca *et al*., (2006)	16 patients (7 with aura, 9 without aura); 15 age- and gender-matched healthy controls	Migraine	• Reduced gray matter density was found bilaterally in dorsolateral prefrontal cortex, ventrolateral prefrontal cortex, temporal cortex, and anterior cingulate cortex • Patients had an increased gray matter volume in PAG compared to healthy controls
Valfré *et al*., (2008)	27 patients, 27 age- and gender-matched controls	Migraine	• In comparison with controls, migraineurs had significant focal gray matter reduction in the right superior temporal gyrus, right inferior frontal gyrus, and left precentral gyrus. • Chronic migraine patients, compared to episodic, showed a focal gray matter decrease in the bilateral anterior cingulate cortex, left amygdala, left parietal operculum, left middle and inferior frontal gyrus, right inferior frontal gyrus, and bilateral Insula. • Significant correlation between gray matter reduction in anterior cingulate cortex and frequency of migraine attacks was found
Kuchinad *et al*., (2007)	10 female patients, 10 age- and gender-matched controls	Fibromyalgia	• Fibromyalgia patients showed a reduction in gray matter volume of left insular cortex, medial prefrontal cortex , left parahippocampal gyrus, and posterior cingulate cortex compared with healthy controls • Gray matter volumes were negatively correlated with duration of fibromyalgia
Schmidt-Wilcke *et al*., (2007)	20 patients, 22 age-matched controls	Fibromyalgia	• Compared to healthy controls, fibromyalgia patients showed a significant decrease in grey matter in the right superior temporal gyrus and left dorsal thalamus • Significant increase in grey matter was found in the left cerebellum in the striatum bilaterally, and the left orbitofrontal cortex in patients compared to healthy controls • There was no significant correlation between pain duration and grey matter values
